# Notification that new names of prokaryotes, new combinations, and new taxonomic opinions have appeared in volume 74, part 8 of the IJSEM

**DOI:** 10.1099/ijsem.0.006534

**Published:** 2024-11-27

**Authors:** Aharon Oren, Markus Göker

**Affiliations:** 1 The Institute of Life Sciences, The Hebrew University of Jerusalem, The Edmond J. Safra Campus, 9190401 Jerusalem, Israel; 2 Leibniz Institute DSMZ – German Collection of Microorganisms and Cell Cultures, Inhoffenstrasse 7B, 38124 Braunschweig, Germany

This listing of names of prokaryotes published in a previous issue of the *International Journal of Systematic and Evolutionary Microbiology* (IJSEM) is provided as a service to microbiology to assist in the recognition of new names and new combinations. This procedure was proposed by the Judicial Commission [1]. The names given herein are listed according to the Rules of priority (i.e., time and date of publication of the articles on the journal’s platform and the order of valid publication of names in the original articles) [2].

**Table:** 

Order of article publication	Name/authors	Proposed as	Article no.	Page no.
1	*Microbacterium humicola* Lee *et al*. 2024	sp. nov.	006486	12
1	*Microbacterium terrisoli* Lee *et al*. 2024	sp. nov.	006486	12
1	*Paenibacillus pedocola* Lee *et al*. 2024	sp. nov.	006486	15
1	*Paenibacillus silviterrae* Lee *et al*. 2024	sp. nov.	006486	15
1	*Flavobacterium terrisoli* Lee *et al*. 2024	sp. nov.	006486	16
1	*Aquabacterium humicola* Lee *et al*. 2024	sp. nov.	006486	16
2	*Gilvimarinus gilvu*s Sun *et al*. 2024	sp. nov.	006490	6
2	*Gilvimarinus algae* Sun *et al*. 2024	sp. nov.	006490	7
3	*Vagococcus jeotgali* Joe *et al*. 2024	sp. nov.	006489	7
4	*Sphingobacterium zhuxiongii* Tao *et al*. 2024	sp. nov.	006488	6
4	*Sphingobacterium luzhongxinii* Tao *et al*. 2024	sp. nov.	006488	8
5	*Psychrosphaera algicola* Bayburt *et al*. 2024	sp. nov.	006491	12
5	*Paraglaciecola algarum* Bayburt *et al*. 2024	sp. nov.	006491	12
6	*Pseudomonas fragariae* Marin *et al*. 2024	sp. nov.	006476	7
7	*Shewanella scandinavica* Martín-Rodríguez *et al*. 2024	sp. nov.	006480	12
7	*Shewanella vaxholmensis* Martín-Rodríguez *et al*. 2024	sp. nov.	006480	12
8	*Dolosigranulum savutiense* Popowitch *et al*. 2024	sp. nov.	006498	8
9	*Nicoliella lavandulae* Alcántara *et al*. 2024	sp. nov.	006497	6
10	*Oxyplasma* Golyshina *et al*. 2024	gen. nov.	006499	8
10	*Oxyplasma meridianum* Golyshina *et al*. 2024	sp. nov.	006499	8
11	*Mycovorax* Thai *et al*. 2024	gen. nov.	006496	8
11	*Mycovorax composti* Thai *et al*. 2024	sp. nov.	006496	8
12	'*Candidatus* Phytoplasma vignae' Rodrigues Jardim *et al*. 2024*	sp. nov.	006502	5
13	*Saeziaceae* Lu and Chen 2024	fam. nov.	006495	9
13	*Pseudaquabacterium* Lu and Chen 2024	gen. nov.	006495	10
13	*Ideonella margarita* Lu and Chen 2024	sp. nov.	006495	10
13	*Ideonella lacteola* Lu and Chen 2024	sp. nov.	006495	10
13	*Pseudaquabacterium inlustre* Lu and Chen 2024	sp. nov.	006495	10
13	*Pseudaquabacterium rugosum* Lu and Chen 2024	sp. nov.	006495	11
13	*Pseudaquabacterium pictum* (Hirose *et al*. 2020) Lu and Chen 2024	comb. nov. [basonym*: Aquabacterium pictum* Hirose *et al*. 2020]	006495	11
13	*Pseudaquabacterium terrae* (Dahal *et al*. 2021) Lu and Chen 2024	comb. nov. [basonym*: Aquabacterium terrae* Dahal *et al*. 2021]	006495	11
13	*Piscinibacter terrae* Lu and Chen 2024	sp. nov.	006495	12
13	*Piscinibacter koreensis* (Chaudhary *et al*. 2022) Lu and Chen 2024	comb. nov. [basonym*: Schlegelella koreensis* Chaudhary *et al*. 2022]	006495	12
13	*Kinneretia aquatilis* Lu and Chen 2024	sp. nov.	006495	12
13	*Kinneretia oligotropha* (Pheng *et al*. 2017) Lu and Chen 2024	comb. nov. [basonym*: Paucibacter oligotrophus* Pheng *et al*. 2017]	006495	12
13	*Kinneretia alba* (Park *et al*. 2023) Lu and Chen 2024	comb. nov. [basonym*: Roseateles albus* Park *et al*. 2023]	006495	12
13	*Kinneretia koreensis* (Park *et al*. 2023) Lu and Chen 2024	comb. nov. [basonym*: Roseateles koreensis* Park *et al*. 2023]	006495	12
13	*Caldimonas aquatica* (Chou *et al*. 2006) Lu and Chen 2024	comb. nov. [basonym*: Schlegelella aquatica* Chou *et al*. 2006]	006495	12
13	*Aquariibacter lacus* Lu and Chen 2024	sp. nov.	006495	13
14	*Edaphobacter paludis* Huber *et al*. 2024	sp. nov.	006500	8
15	*Gilvirhabdus* Jayan *et al*. 2024	gen. nov.	006474	7
15	*Gilvirhabdus luticola* Jayan *et al*. 2024	sp. nov.	006474	8
16	*Methylomonas rivi* Abin *et al*. 2024	sp. nov.	006506	10
16	*Methylomonas rosea* Abin *et al*. 2024	sp. nov.	006506	12
16	*Methylomonas aurea* Abin *et al*. 2024	sp. nov.	006506	12
16	*Methylomonas subterranea* Abin *et al*. 2024	sp. nov.	006506	12
17	*Actinomycetospora aeridis* Suriyachadkun *et al*. 2024	sp. nov.	006505	7
17	*Actinomycetospora flava* Suriyachadkun *et al*. 2024	sp. nov.	006505	8
17	*Actinomycetospora aurantiaca* Suriyachadkun *et al*. 2024	sp. nov.	006505	8

*Names of *Candidatus* taxa are not validly published.

The following taxonomic opinions (i.e., the emendation of circumscriptions or the creation of synonyms) do not affect the status of a name under the International Code of Nomenclature of Prokaryotes. These taxonomic opinions can neither be considered as approved nor as disapproved by the International Committee on Systematics of Prokaryotes.

**Table:** 

Name/authors:	Article no.	Page no.
*Kineococcus aureolus* Xu *et al*. 2017 pro synon. *Kineococcus terrestris* Xu *et al*. 2017	006504	4
*Ornithinicoccus soli* Jiang *et al*. 2020 pro synon. *Segeticoccus rhizosphaerae* Lee and Whang 2020	006503	4
*Pseudoalteromonas distincta* (Romanenko *et al*. 1995) Ivanova *et al*. 2000 emend. Bayburt *et al*. 2024	006491	13
*Pseudoalteromonas elyakovii* (Ivanova *et al*. 1987) Sawabe *et al*. 2000 pro synon. *Pseudoalteromonas distincta* (Romanenko *et al*. 1995) Ivanova *et al*. 2000	006491	11
*Pseudoalteromonas flavipulchra* Ivanova *et al*. 2002 pro synon. *Pseudoalteromonas maricaloris* Ivanova *et al*. 2002	006491	11
*Pseudoalteromonas gelatinilytica* Yan *et al*. 2016 emend. Bayburt *et al*. 2024	006491	13
*Pseudoalteromonas maricaloris* Ivanova *et al*. 2002 emend. Bayburt *et al*. 2024	006491	13
*Pseudoalteromonas profundi* Zhang *et al*. 2016 pro synon. *Pseudoalteromonas gelatinilytica* Yan *et al*. 2016	006491	11
*Williamsia marianensis* Pathom-aree *et al*. 2006 pro synon. *Williamsia muralis* Kämpfer *et al*. 1999	006485	5
*Williamsia muralis* Kämpfer *et al*. 1999 emend. Kumar *et al*. 2024	006485	5
